# Turkish Adaptation and Validation of the EMpowerment of PArents in THe Intensive Care (EMPATHIC-30) Questionnaire to Measure Parent Satisfaction in Neonatal Intensive Care Units

**DOI:** 10.3389/fped.2020.00421

**Published:** 2020-07-29

**Authors:** Öznur Tiryaki, Hamide Zengin, Nursan Çınar, Mümtaz Mutlu Umaroǧlu, Jos M. Latour

**Affiliations:** ^1^Institute of Health Sciences, Sakarya University, Sakarya, Turkey; ^2^Department of Pediatric Nursing, Faculty of Health Sciences, Bilecik Şeyh Edebali University, Bilecik, Turkey; ^3^Department of Pediatric Nursing, Faculty of Health Sciences, Sakarya University, Sakarya, Turkey; ^4^Faculty of Medicine Basic Medical Sciences, Biostatistics, Sakarya University, Sakarya, Turkey; ^5^Faculty of Health: Medicine, Dentistry and Human Sciences, School of Nursing and Midwifery, University of Plymouth, Plymouth, United Kingdom

**Keywords:** EMPATHIC-30, parents, infants, satisfaction, neonatal intensive care unit, reliability, validity

## Abstract

**Aim:** The aim of this study was to translate and validate the shortened version of the “EMpowerment of PArents in THe Intensive Care” (EMPATHIC-30) questionnaire into Turkish to measure parent satisfaction in the Neonatal Intensive Care Units (NICU).

**Method:** The study used a cross-sectional design. The data of the study were collected from parents with infants staying in the NICU of a training and research hospital in Sakarya, Turkey, between July 2018–2019 after obtaining ethical approval. Totally, 238 parents (234 mothers, four fathers) agreed to participate in the study and completed the questionnaire. Of these, 35 mothers were recruited 1 month later for the test-retest reliability analysis. The questionnaire was translated using back and forward translation. Reliability and validity test were performed to measure the psychometric properties of the Turkish EMPATHIC-30.

**Results:** The mean age of the parents was 28.27 (SD 5.93), and 48.3% of them were primary school graduates. The infants: 55.9% were male, the mean gestational age was 36.89 (SD 3.25) weeks, and mean length of hospital stay was 9.36 (SD 10.17) days. The mean scores of each item with a six-point scale of the EMPATHIC-30 questionnaire ranged between 4.01 and 4.87. The Cronbach's alpha of the total questionnaire was 0.95. Cronbach's alpha of the five domains (Information, Care and Treatment, Organization, Parent Participation, and Professional Attitude) ranged between 0.80 and 0.92. Pearson correlation coefficient between the domains and total questionnaire was *r* = 0.988. The Intraclass correlation coefficient was ICC = 0.998 in the test-retest evaluation. Confirmatory factor analysis was performed for construct validity and was moderate; Comparative Fit Index = 0.792, Tucker–Lewis Index = 0.770, Standardized Root Mean Square Residual = 0.0811, and Root Mean Square Error of Approximation = 0.107.

**Conclusion:** The Turkish version of EMPHATIC-30 has adequate psychometric properties. The EMPATHIC-30-Turkish questionnaire is an easy and appropriate instrument which can be used to measure satisfaction of Turkish parents with infants staying in the NICU.

## Introduction

The hospitalization of an infant in a neonatal intensive care unit (NICU) is a stressful situation for both parents and the infant (1). This may affect the family's daily routines and may lead to changes in their roles and responsibilities in the family environment (2). Besides these changes in the family environment, not knowing the NICU environment, encountering medical devices, and changing duties in the care of their infant can cause anxiety and fear among parents and family members (3).

Family-Centered Care (FCC) interventions have been developed and implemented to minimize stress and anxiety experienced by parents and accelerate the healing process of infants (4, 5). An important element in maintaining the FCC approach is effective communication. Furthermore, developing mutual trust, reducing conflict, minimizing stress levels of parents and improving parental satisfaction are the components of FCC (6–8). It is known that parents whose have experienced an admission of their infant in the NICU need information on many issues during admission, at discharge and when at home after hospital discharge. These information needs can be clustered in five themes: communication, parental role clarity, emotional support, information resources, and financial resources (9). Studies exploring problems of parents with premature infants in the NICU identified that parents had difficulties in bonding with their infant, breastfeeding, were worried about being away from their infant and had difficulties with information and communication with healthcare professionals (10, 11).

Implementing the principles of FCC can reduce hospital length of, improve the bonding between parents and infants, and increase parent satisfaction (8, 12). Parental satisfaction is used as an indicator to measure quality of care and the use of satisfaction surveys is an effective method for evaluating health services (13). Currently, there are no validated parent satisfaction instruments available for parents in Turkish NICUs. Although the 65-item EMPATHIC-N has been developed and tested specifically for parents in the NICU, there is no short version of this questionnaire (14). To reduce the burden of parents we preferred to have a shorter version and therefore we used the short version of the EMPATHIC questionnaire, the EMPATHIC-30 (14, 15). Therefore, the aim of this study was to translate, culturally adapt, and validate the EMPATHIC-30 questionnaire to measure parent satisfaction in Turkish NICUs.

## Materials and Methods

### Design

The study used a cross-sectional descriptive design. Ethical approval was obtained from the hospital research ethics committee (02/04/2018–72) and written consent was obtained from the parents who participated in the study. Data collection was performed between July 2018 and July 2019.

### Setting

The study setting was at the NICU of the Sakarya Training and Research Hospital in the west of Turkey. The tertiary NICU serves the province of Sakarya. The NICU has a capacity of 29 beds: one level III unit with 18 beds, one level II unit with nine beds, and one level I unit with six beds. Annual admission rate of the NICU is around 900 infants. The level III unit admits infants with a birth weight <1,500 grams, the level II unit admits infants with a birth weight between 1,500 and 2,500 grams and the level I unit has infants >2,500 grams.

### Participants

The study sample consisted of 238 parents (234 mothers, four fathers) who agreed to participate in the study. Of the study participants, 35 mothers agreed to be interviewed for the test-retest of the Turkish EMPATHIC-30 questionnaire. Mothers or fathers were included in the study if their infants stayed in the NICU for at least 2 days, volunteered to participate in the study, provided written consent, and speak and understand Turkish.

### EMPATHIC-30 Questionnaire

The EMPATHIC-30 questionnaire consists of three parts (16). The first part includes demographic questions such as parent's age, education level, income level, number of children in the family, and working status. Furthermore, mother's type of birth, number of pregnancies, birth/postpartum problem status and characteristics of the infants such as infant's gender, gestational week, days of NICU stay, and feeding type have been added. The second part of the EMPATHIC-30 questionnaire included the 30 items divided in five domains. The short version of the EMPATHIC-30 questionnaire was developed from the EMPATHIC initial version (15). This version was developed in eight Pediatric Intensive Care Units (PICU) in the Netherlands (15). Further statistical redundancy testing with 3,354 parents resulted in the short version of the EMAPTHIC-30 questionnaire (16). The EMPATHIC-30 consists of five domains: Information (five items), Care & Treatment (eight items), Organization (five items), Parent Participation (six items), and Professional Attitude (six items). The answer option scale is a 6-point Likert type (1 = certainly no; 6 = certainly yes) and each item has an additional “Not Applicable” option. The reliability estimates (Cronbach's α) of the domains were adequate and ranged from 0.73 to 0.93. Approval to use the EMPATHIC-30 was granted by the developer (J.M. Latour) and was part of the research team.

### Translation and Cultural Adaptation

The translation and cultural adaptation process followed the Principles of Good Practice for the Translation and Cultural Adaptation of Patient-Reported Outcomes Measures described by the task force of the International Society for Pharmacoeconomics and Outcomes Research (17). This 10-step process included:

Step 1: Preparation: Permission was granted by the developer to use EMPATHIC-30 questionnaire. The EMAPTHIC-30 was revised before translation; PICU or ICU was reworded to NICU.
Step 2: Forward translation: The translation was performed in Turkish language by two translators independently. With approval of the developer, the EMATHIC-30-UK was used for the translation.
Step 3: Reconciliation: The translated version was reviewed on meaning and spelling of the items by PICU and research experts.
Step 4: Back translation: The Turkish EMPATHIC-30 was translated back to English by one translator.
Step 5: Back translation review: The translation was found to be sufficiently translated to the original questionnaire.
Step 6: Harmonization: The questionnaire was reviewed by the research team and the developer and was found to be suitable for the Turkish population.
Step 7: Cognitive debriefing: The Turkish EMPATHIC-30 was reviewed by nine experts in Child Health and Diseases Nursing Department, Department of Child Health and Diseases, Department of Child Psychiatry, and Internal Medicine Nursing Department. The experts were asked to assess the suitability and clarity of each item. They were asked to rate each statement between 1 and 4 points (one point: not appropriate, two points: slightly appropriate, three points: appropriate, four points: completely appropriate), and write their opinions and suggestions for each item.
Step 8: Review of cognitive debriefing results and finalization: In line with the opinions of the experts, the items were reviewed, and necessary changes were made. As a result of the evaluation of experts, all items were corrected in terms of language and expression with the suggestions and contributions of experts.
Step 9: Proofreading: The Turkish EMPATHIC-30 questions were reviewed. After applying the final version of the scale to 10 parents, it was decided that there were no unclear expressions.
Step 10: Final Report: This paper presents the final report and further validity testing.

### Data Collection

The data of the study were collected from parents when their infants were discharged from the NICU. Data collection forms were given to parents and asked to complete.

### Data Analysis

Number and percentage (*n*, %) were used to define categorical variables in order to identify the characteristics of the data of 238 participants; mean and standard deviation was used to define numerical variables. It was planned to use a minimum of 10% sample for the test-retest (18). The test-retest reliability of the questionnaire was performed with 35 parents after 1 month and correlation coefficient (r) and intraclass correlation coefficient (ICC) were examined for test-retest reliability. Confirmatory factor analysis (CFA) is tailored to unraveling the empirical structure of the interrelationship of the 30 statements. The final model was based on both theoretical and statistical plausibility. The measures applied in this study were v2 test of model fit, and the ratio of v2 df \3 represents a good model fit. Other tests used for the model fit were: comparative fit index (preferably CFI C 0.95), Tucker–Lewis index (preferably TLI C 0.95), root mean square error of approximation (preferably RMSEA \0.08), and the weighted root mean square residual (preferably WRMR\0.90) (19). Data were evaluated with the statistical software program IBM SPSS Statistics 22 and type 1 error (α) was set at 0.05.

## Results

Of the 238 parents who returned the questionnaire, 98.3% were mothers with a mean age 28.27 (SD 5.93). Furthermore, 48.3% were primary school graduates, 21% were employed, and 44.5% had moderate income. According to pregnancy histories, 38.1% mother had their first baby, 78.1% had pregnancy planned, 95.8% had spontaneous pregnancy, 42.9% had normal birth, 42.9% had at least one child. 55.9% of the infants were male with a mean gestational age of 36.89 (SD 3.25) weeks and the mean birth weight was 2863.82 (SD 238.03) grams. Hospital length of stay was 9.36 (SD 10.17) days. During the hospital stay, 56.7% of the infants received only breast milk, 42.4% received both breast milk and formula (Table 1).

**Table 1:** Descriptive characteristics of parents and infants.

**Characteristics**	***N* = 238**	**%**
**Participant**		
Mother	234	98.3
Father	4	1.7
**Education**		
Primary education	115	48.3
High school	79	33.2
License	49	16.8
Postgraduate	4	1.7
**Working condition**		
Yes	50	21
No	188	79
**Income status (family's own statement)**		
Good	55	23
Middle	106	44.5
Bad	77	32.5
**Gender of infant**		
Girl	105	44.1
Male	133	55.9
**Number of children**		
1	102	42.8
2	73	30.7
≥3	63	26.5
**Person taken information related to infant**		
Doctor	205	86.1
Nurse	24	10.1
Doctor and Nurse	5	2.1
Medical secretary	4	1.7
**Total pregnancy number of mother**		
1	92	38.7
2	64	26.9
3	51	21.4
4	13	5.4
≥ 5	18	7.6
**Birth type**		
Normal	102	42.9
Cesarean	136	57.1
**Planned pregnancy**		
Yes	174	73.1
No	64	26.9
**Infant feeding type**		
Breast milk only	135	56.8
Breast milk and formula	101	42.4
Only formula	2	0.8
Age parents (mean, SD)	28.27 (5.93)	Min–max: 15-48
Infant gestational age in weeks	36.89 (3.25)	Min–max: 26-41
(mean, SD)		
Infant's birth weight (mean, SD)	2863.82 (238.03)	Min–max: 630-5800
Length of stay NICU in days (mean, SD)	9.36 (10.17)	Min–max: 2-78

*SD, Standard Deviation*.

The Cronbach's alpha of the domains of the EMPATHIC-30 questionnaire ranged from 0.804 to 0.922 (Table 2). The mean scores and standard deviations are presented in Table 2. The mean score, standard deviation, and total Cronbach's alpha coefficient of each item are presented in Table 3. The lowest mean score was the item ‘We received understandable information about the effects of the medication’ (mean 4.01, SD 1.40). The highest rated item was “The team respected the privacy of our child's and of us” (mean 4.81, SD 0.89).

**Table 2:** Mean (SD) and Cronbach's α of the domains of the Turkish EMPATHIC-30.

**Domains**	**Mean**	**Standard deviation**	**Min**	**Max**	**Cronbach alpha**
1. Information	21.87	4.54	8	30	0.831
2. Care & treatment	35.98	6.36	20	48	0.848
3. Organization	22.54	4.22	9	30	0.804
4. Parent participation	27.69	4.71	15	36	0.869
5. Professional attitude	27.85	5.10	9	36	0.922

**Table 3:** Descriptive analysis EMPATHIC-30 items.

**EMPATHIC-30 items**	**Mean**	**Standard deviation**	**Cronbach's alpha if item deleted**
1. We had daily talks about our child's care and treatment with the doctors	4.59	1.04	0.953
2. We had daily talks about our child's care and treatment with the nurses	4.44	1.14	0.953
3. The doctor clearly informed us about the consequences of our child's treatment	4.56	1.08	0.952
4. We received clear information about the examinations and tests	4.27	1.19	0.952
5. We received understandable information about the effects of the medication	4.01	1.40	0.954
6. The doctors and nurses worked closely together	4.74	0.91	0.952
7. We were well-prepared for our child's discharge by the doctors	4.22	1.48	0.955
8. We were well-prepared for our child's discharge by the nurses	4.31	1.40	0.955
9. The team was alert to the prevention and treatment of pain in our child	4.75	0.80	0.952
10. Our child's comfort was taken into account by the doctors	4.80	0.83	0.952
11. Our child's comfort was taken into account by the nurses	4.76	0.87	0.953
12. Every day we knew who was responsible for our child, regarding the doctors	4.12	1.32	0.952
13. Every day we knew who was responsible for our child, regarding the nurses	4.27	1.28	0.952
14. The team worked efficiently	4.64	1.01	0.952
15. The IC-unit could easily be reached by telephone	4.31	1.28	0.955
16. There was enough space around our child's bed	4.45	1.21	0.953
17. The IC-unit was clean	4.69	0.95	0.952
18. Noise in the IC-unit was muffled as good as possible	4.45	1.15	0.952
19. During our stay the staff regularly asked for our experiences	4.41	1.13	0.952
20. We were actively involved in decision-making on care and treatment of our child	4.47	1.09	0.951
21. We were encouraged to stay close to our child	4.72	0.93	0.952
22. We had confidence in the doctors	4.87	0.77	0.952
23. We had confidence in the nurses	4.87	0.79	0.952
24. Even during intensive procedures we could always stay close to our child	4.36	1.25	0.953
25. We received sympathy from the doctors	4.48	1.14	0.952
26. We received sympathy from the nurses	4.49	1.12	0.952
27. The team worked hygienically	4.72	0.92	0.952
28. The team respected the privacy of our child's and of us	4.81	0.89	0.952
29. The team showed respect for our child and for us	4.80	0.85	0.952
30. At admission we felt welcome	4.55	1.05	0.952

The Pearson correlation coefficient (r) with the total score of each domain ranged from 0.806 to 0.900 (Table 4). One month after the test, 35 parents completed the retest questionnaire and the Pearson correlation coefficient between the two evaluations was *r* = 0.988; Intraclass correlation coefficient was ICC = 0.998. Confirmatory factor analysis for the construct validity confirmed a moderate model fit with the preferred values of Comparative Fit Index and the Tucker-Lewis Index slightly below the preferred ≥0.95. The Root Mean Square Error of Approximation (preferably <0.08) was 0.107 while the Standardized Root Mean Square Residual RMSEA was adequate performed with 0.081, preferably <0.90 (Table 5).

**Table 4:** Correlations of domains and total EMPATHIC-30.

**Domains**	**Total**
1. Information	0.806
2. Care & treatment	0.900
3. Organization	0.847
4. Parent participation	0.874
5. Professional attitude	0.861

**Table 5:** Compliance index values of measurement model.

**EMPATHIC-30 items**	**Compliance index values**
CFI	0.792
TLI	0.770
RMSEA	0.107
SRMR	0.081

*CFI, Comparative Fit Index; TLI, Tucker–Lewis Index; SRMR, Standardized Root Mean Square Residual; RMSEA, Root Mean Square Error of Approximation*.

## Discussion

Family-centered care practices not only increase parental satisfaction but also improve the quality of care (20). A search of the literature revealed that there are no questionnaires available to measure parent satisfaction among Turkey parents in the NICU. The EMPATHIC-30 questionnaire is widely used in many countries to measure family satisfaction (21–23). Our findings are in line with previous investigations of the adaptation of EMPATHIC-30 in other languages. It seems that the EMPATHIC-30 instrument can be applied to measure parental satisfaction and can be adapted in different cultural and linguistic backgrounds.

As a result of the analysis, the Cronbach's alpha values in our study of the five domains (between 0.804 and 0.922) showed that the reliability levels of the questionnaire are high. In the original study of the shortened EMPATHIC-30 study, the Cronbach's alpha values ranged between 0.73 and 0.81 (16). Other studies translating and validating the EMAPTHIC-30 in Italy, Spain and Brazil reported similar internal consistency figure compared to the original study (16, 22–24). Surprisingly, a study in South Africa reported much lower Cronbach's alpha on domain levels; between 0.25 and 0.59 (21). The authors addressed these differences because of a limitation of their small study sample of 100 parents influencing the scores (21).

The means of all items in the EMPATHIC-30 in our study were all below five. This is in contrast with all other similar studies (16, 21–25). The study in South Africa is the only study reporting that all items had a mean score above five (21). This might indicate that parent satisfaction is culturally dependent. However, another explanation could be the family-centered care practices that may vary across countries. In our study and setting it can be argued that family-centered care is not yet fully implemented and therefore parents might have rated the satisfaction items lower as reported in other countries. Further studies are needed to explore the relationship between different family-centered care practices and parent satisfaction outcomes (26, 27).

In our NICU, parents whose infants are hospitalizing generally take the role of visitors while their babies are cared for and treated by nurses and doctors. However, when parents are accepted as members of the healthcare team and value their own knowledge and skills, they feel more adequate and safer in caring for their infants (28, 29). In this case, it becomes easier to deal with changes in the family role. Although our study is important in terms of revealing that the satisfaction of parents in this regard is not at the desired level, we think that this questionnaire, which has been adapted in Turkish will increase the interest in the subject and contribute to improving family-centered care practices in Turkish NICUs.

The highest rated domain of the Turkish EMPATHIC-30 questionnaire was the domain of “Professional Attitude.” This was comparable with the EMPATHIC-30 AUS study in Australia, while other similar studies from Italy, Brazil and Greek-Cyprus demonstrated the highest mean values in the domain “Care and Treatment” (12, 23–25). Variables such as functioning of the international health system, the NICU conditions and the demographic characteristics of parents and their expectations might affect their perceptions of health. The high level of trust in nurses and doctors in our study increased the value of 'Professional Attitude'. However, and overall, our findings indicate that a revisit of our family-centered care practices is needed.

Our study warrants some limitations. First, the number of fathers participating in the study was low. This might be because in our culture, fathers are often working during the daytime and have limited time to visit the NICU while also caring for siblings and other socio-economic issues. Another limitation is the translation process. Although agreed by the developer, we did not use the original Dutch version but instead the translated English version which has been used in the UK. Finally, we acknowledge that our study included only parents from one NICU. Further multi-center testing would be needed and could enhance the acceptability of the validated Turkish version of the EMPATHIC-30 instrument.

## Conclusion

Based on the results of our study, the Turkish version of EMPATHIC-30 is a reliable and valid instrument that can be used to measure satisfaction of the parents in NICU settings. The EMPATHIC-30 Turkish can be considered as a benchmark tool to learn from the parental reported outcomes of other NICUs. Finally, the instrument can be used among parents from Turkish origin in other countries.

## Data Availability Statement

The raw data supporting the conclusions of this article will be made available by the authors, without undue reservation, to any qualified researcher.

## Ethics Statement

The studies involving human participants were reviewed and approved by Sakarya University Ethics Committee. The patients/participants provided their written informed consent to participate in this study.

## Author Contributions

ÖT contributed to the design of the study, contributed to data collection, data analysis and interpretation, and drafted the first manuscript. HZ, NÇ, and MU contributed to data collection, data analysis and interpretation, and provided revisions to early drafts. JL contributed to the design of the study and drafted the first manuscript. All authors contributed, read, and approved the final manuscript.

## Conflict of Interest

The authors declare that the research was conducted in the absence of any commercial or financial relationships that could be construed as a potential conflict of interest.

## References

[1] Yayan EH Özdemir M Düken ME Suna Dag Y Determination of stress levels of parents in newborn intensive care unit of baby. GÜSBD. (2019) 8:82–9.

[2] Çirlak A Erdemir F Comfort level of parents who have newborns in neonatal intensive care units. Anadolu Hemşirelik ve Saglik Bilimleri Dergisi. (2013) 16:73–81.

[3] Boztepe H Çavuşoglu H Examination of a family centered care practice at the children's units of a university hospital. Hacettepe Univ Facul Health Sci Nurs J. (2009) 16:11–24.

[4] Zhang R Huang RW Gao XR Peng XM Zhu LH. Involvement of parents in the care of preterm infants: a pilot study evaluating a family-centered care intervention in a chinese Neonatal ICU. Pediatr Crit Care Med. (2018) 19:741–7. 10.1097/PCC.0000000000001586 29781955

[5] Lv B Xi-ronga G Jing S Tao-tao L Zhen-ye L. Family-centered care improves clinical outcomes of very-low-birth-weight infants: a quasi-experimental study. Front Pediatr. (2019) 7:138. 10.3389/fped.2019.00138 31032240 PMC6473064

[6] Zurca AD Corriveau CO. Family communication in the PICU: Less than ideal no matter how you say it. Crit Care Med. (2014) 42:1569–70. 10.1097/CCM.0000000000000255 24836804

[7] Zurca AD Fisher KR Flor RJ. Communication with limited English-proficient families in the PICU. Hosp Pediatr. (2017) 7:9–15. 10.1542/hpeds.2016-0071 27979992 PMC5740871

[8] Ding X Zhu LH Zhang R Wang L Wang TT Latour JM. Effects of family-centered care interventions on preterm infants and parents in neonatal intensive care units: a systematic review and meta-analysis of randomized controlled trials. Austral Crit Care. (2019) 32:63–75. 10.1016/j.aucc.2018.10.007 30554939

[9] Berman L Raval MV Ottosen M Mackow AK Cho M Goldin AB. Parent perspectives on readiness for discharge home after neonatal intensive care unit admission. J Pediatr. (2019) 205:98–104. 10.1016/j.jpeds.2018.08.086 30291021

[10] Russell G Sawyer A Rabe H Abbott J Gyte G Duley L. Parents' views on care of their very premature babies in neonatal intensive care units: a qualitative study. BMC Pediatr. (2014) 14:230–240. 10.1186/1471-2431-14-230 25216714 PMC4190336

[11] Balci S Yildirim Balkan Z Yenidoǧan yoǧun bakım ünitesinde aile merkezli bakım. Türkiye Klinikleri Çocuk Saǧliǧi ve Hastaliklari Hemşireliǧi-Özel Konular. (2019) 2:18–23.

[12] Papamichael E Ioannou M Talias MA. EMPATHIC-N in a Greek-Cypriot sample: confirming its factorial structure. BMC Health Serv Res. (2018) 18:968. 10.1186/s12913-018-3793-3 30547797 PMC6295023

[13] Weissenstein A Straeter A Villalon G Luchter E Bittman S. Parent satisfaction with a pediatric practice in Germany: a questionnaire-based study. Ital J Pediatr. (2011) 37:31–37. 10.1186/1824-7288-37-31 21729322 PMC3163525

[14] Latour JM Duivenvoorden HJ Hazelzet JA van Goudoever JB. Development and validation of a neonatal intensive care parent satisfaction instrument. Pediatr Crit Care Med. (2012) 13:554–9. 10.1097/PCC.0b013e318238b80a 22460771

[15] Latour JM van Goudoever JB Duivenvoorden HJ Albers MJIJ van Dam NAM Dullaart E. Construction and psychometric testing of the EMPATHIC questionnaire measuring parent satisfaction in the pediatric intensive care unit. Intensive Care Med. (2011) 37:310–8. 10.1007/s00134-010-2042-y 20848078 PMC3028088

[16] Latour JM Duivenvoorden HJ Tibboel D Hazelzet JA the EMPATHIC Study Group. The shortened EMpowerment of PArents in THe Intensive Care 30 questionnaire adequately measured parent satisfaction in pediatric intensive care units. J Clin Epidemiol. (2013) 66:1045–50. 10.1016/j.jclinepi.2013.02.010 23790723

[17] Wild D Grove A Martin M Eremenco S McElroy S Verjee-Lorenz. ISPOR task force for translation and cultural adaptation. Principles of good practice for the translation and cultural adaptation process for patient-reported outcomes (PRO) measures: report of the ISPOR Task Force for translation and cultural adaptation. Value Health. (2005) 8:94–104. 10.1111/j.1524-4733.2005.04054.x 15804318

[18] Arli M Nazik H Bilimsel Araştirmaya Giriş. Ankara: Gazi Kitabevi (2001). p. 77

[19] Schreiber JB. Core reporting practices in structural equation modeling. Res Soc Adm Pharm. (2008) 4:83–97. 10.1016/j.sapharm.2007.04.003 18555963

[20] Meert KL Clark J Eggly S. Family-centered care in the pediatric intensive care unit. Pediatr Clin North Am. (2013) 60:761–72. 10.1016/j.pcl.2013.02.011 23639667 PMC3767974

[21] Mol C Argent AC Morrow BM Parental satisfaction with the quality of care in a South African paediatric intensive care unit. S Afr J Crit Care. (2018) 34:50–6. 10.7196/SAJCC.2018.v34i2.366

[22] Orive FJP Lozano JB Zuñiga AL Fernández YML Argaluza JE Latour JM. Spanish translation and validation of the EMPATHIC-30 questionnaire to measure parental satisfaction in intensive care units. An Pediatr (Barc). (2018) 89:50–7. 10.1016/j.anpede.2017.08.006 29108859

[23] Dall'Oglio I Fiori M Tiozzo E Mascolo R Portanova A Gawronski O. Neonatal intensive care parent satisfaction: a multicenter study translating and validating the Italian EMPATHIC-N questionnaire. Ital J Pediatr. (2018) 44:5. 10.1186/s13052-017-0439-8 29304879 PMC5756347

[24] Gomez DBCA Vidal SA Lima LCS. Brazilian adaptation and validation of the Empowerment of Parents in the Intensive Care-Neonatology (EMPATHIC-N) questionnaire. J Pediatr (Rio J.). (2017) 93:156–64. 10.1016/j.jped.2016.06.007 27565641

[25] Gill FJ Wilson S Aydon L Leslie GD Latour JM. Empowering parents of australian infants and children in hospital: translation, cultural adaptation, and validation of the empowerment of parents in the intensive care-30-AUS questionnaire. Pediatr Crit Care Med. (2017) 18:e506–13. 10.1097/PCC.0000000000001309 28906423

[26] Dall'Oglio I Mascolo R Tiozzo E Portanova A Fiori M Gawronski O. The current practice of family-centred care in Italian neonatal intensive care units: a multi-centre descriptive study. Intensive Crit Care Nurs. (2019) 50:36–43. 10.1016/j.iccn.2018.07.005 30075992

[27] Lake ET Smith JG Staiger DO Hatfield LA Cramer E Kalisch BJ. Parent satisfaction with care and treatment relates to missed nursing care in Neonatal Intensive Care Units. Front Pediatr. (2020) 8:74. 10.3389/fped.2020.00074 32257979 PMC7093579

[28] Segers E Ockhuijsen H Baarendse P van Eerden I van den Hoogen A The impact of family centred care interventions in a neonatal or paediatric intensive care unit on parents' satisfaction and length of stay: a systematic review. Intensive Crit Care Nurs. (2019) 50:63–70. 10.1016/j.iccn.2018.08.008 30249426

[29] Kamphorst K Brouwer AJ Poslawsky IE Ketelaar M Ockhuisen H van den Hoogen A. Parental presence and activities in a dutch neonatal intensive care unit: an observational study. J Perinat Neonatal Nurs. (2018) 32:E3–E10. 10.1097/JPN.0000000000000354 30036311

